# Oral mucosal health in acromegaly: clinical insights and metabolic associations

**DOI:** 10.1186/s12903-025-07421-0

**Published:** 2025-12-07

**Authors:** Burak Can Cengiz, Sefika Burcak Polat, Elif Yildizer, Ihsan Ates, Reyhan Ersoy, Oya Topaloglu, Bekir Cakir

**Affiliations:** 1 Department of Internal Medicine, Ankara City Hospital, Ankara, Turkey; 2 Department of Endocrinology & Metabolism, Ankara City Hospital, Ankara, Turkey; 3 Department of Oral, Dental and Maxillofacial Radiology, Ankara Yildirim Beyazit University, 15 Temmuz Şehitleri Binası, Ayvalı Mah. 150. Sk. Etlik-Keçiören , Ankara, Turkey

**Keywords:** Acromegaly, Oral mucosal lesions, Dental hygiene

## Abstract

**Objectives:**

This study aimed to evaluate oral mucosal lesions, dental status, and metabolic associations in patients with acromegaly, and to explore the impact of treatment and comorbidities.

**Materials and methods:**

This prospective case-control study included acromegaly patients and healthy controls. Oral health assessment was performed using the DMFT index, the Periodontal Screening Index (PSI), and the Plaque Index (PI).Oral hygiene practices were assessed through a structured questionnaire. Laboratory parameters, including GH, IGF-1, ACTH, cortisol, TSH, T4, fasting glucose, and HbA1c, were measured. Statistical analysis included group comparisons and correlation assessments with significance set at *p* < 0.05.

**Results:**

There were no significant differences between groups in oral hygiene scores, DMFT values, PSI, or PI scores (*p* > 0.05). However, macroglossia (82.9%) and fissured tongue (51.4%) were significantly more common in patients with acromegaly (*p* < 0.001). In the acromegaly group, no significant associations were found between DMFT scores and clinical or hormonal parameters and a negative correlation was observed between oral hygiene and PSI scores (*p* < 0.05), and a positive correlation between PI and PSI scores (*p* < 0.001). No significant correlations were observed in the control group.

**Conclusion:**

Tongue lesions were significantly more prevalent in patients with acromegaly, regardless of disease status or treatment. While general oral health parameters were similar between groups, periodontal inflammation in acromegaly was linked to poor oral hygiene. These findings suggest that acromegaly may be a risk factor for periodontal disease. Routine oral health monitoring is essential in this population.

## Introduction

Acromegaly presents as a chronic endocrine disorder characterised by hypersecretion of growth hormone (GH) and insulin-like growth factor-1 (IGF-1), primarily induced by a GH-secreting pituitary adenoma. Predominantly originating in the anterior pituitary gland, these adenomas prompt elevated levels of circulating IGF-1, which serves as the principal mediator of GH actions. Manifestations of the condition manifest gradually, manifesting in multisystemic involvement, resulting in somatic changes, progressive morphological alterations, and a complex spectrum of systemic comorbidities [1, 2]. These somatic alterations often develop slowly, leading to diagnostic delays. Predominantly, early signs of acromegaly include facial dysmorphisms, orodental manifestations, extremity enlargement, and soft tissue hypertrophy [3]. Notably, soft tissue hypertrophy in the craniofacial and cervical regions, along with skeletal overgrowth, particularly evident in the cranium, stand out as prominent features of the disorder [4].

Orofacial findings are common in acromegaly and include mandibular prognathism, diastema, malocclusion, macroglossia, temporomandibular joint pain, and facial dysmorphism [4–7]. The prevalence and progression of these clinical findings across various disease stages (active, remission, and recovery) have been extensively studied [4]. Oral findings are among the earliest signs in approximately 10–20% of individuals and may be present in as many as 80% at diagnosis [4, 8–11]. Craniofacial features such as jaw enlargement, buccal tipping of the mandibular teeth, and enlarged pulp chambers (taurodontism) have also been documented radiographically [1, 5, 7, 12–16]. Although oral tissues are frequently affected, tongue lesions other than macroglossia (e.g., fissured tongue, hairy tongue) and lip conditions other than macrocheilia (e.g., angular cheilitis, actinic cheilitis) have not been comprehensively evaluated in patients with acromegaly.

Patients with acromegaly may experience multiple endocrine deficiencies following surgical interventions or radiotherapy. Hypothyroidism, often associated with pronounced xerostomia [17], can contribute to poor oral hygiene and increased susceptibility to dental caries. Similarly, oral mucosal complications resulting from late-onset diabetes mellitus and the administration of somatostatin receptor ligands, which reduce general secretions as part of disease management ([18]– [19]), can exacerbate dry mouth and related dental issues.

Studies investigating oral and dental health in acromegaly are quite limited and have primarily relied on self-reported data obtained through questionnaires [4, 20]. Clinical data derived from professional dental examinations remain scarce. Survey-based studies have reported orodental symptoms in 37–48% of patients, with the most common complaints being difficulties in eating, speech problems, and dissatisfaction with dental aesthetics. Studies involving intraoral physical examinations have mainly focused on periodontal health [11, 21].

The effects of GH and IGF-1 on periodontal tissues remain controversial. While some studies suggest that gingival overgrowth may predispose individuals to periodontitis, others propose a protective role, possibly due to increased soft tissue volume and thick gingival biotype [21, 22]. When evaluating the impact of acromegaly on dental and periodontal tissues, factors such as patients’ perceived importance of oral health, hygiene behaviours, and the presence of xerostomia should also be taken into consideration.

This study aimed to evaluate oral mucosal lesions and dental health in patients with acromegaly compared to healthy controls. Specifically, we sought to determine the prevalence of soft tissue lesions, assess oral hygiene behaviours and dental status using objective indices (DMFT, PSI, PI), and explore potential associations with biochemical parameters (IGF-1, GH, ACTH, cortisol), disease activity, treatment modalities, and comorbid (xserestomia etc.) conditions. We hypothesised that patients with acromegaly would exhibit a higher prevalence of oral mucosal lesions and compromised dental and periodontal health compared to controls, independent of disease activity or hormonal control.

## Methods

Approval for the prospective case-control study was obtained from the Health Sciences University Ankara City Hospital Ethics Committee for Clinical Research (approval ID: E2-22–2670, November 23, 2022).The study was conducted between November 2022 and April 2023 in the Endocrinology and Metabolism and Internal Medicine departments of Ankara City Hospital.

Adult patients aged 18 years and older diagnosed with acromegaly, either as outpatients or inpatients, were included in the study. Participants in the control group were healthy adults (≥ 18 years), matched by age and gender, with no diagnosis of acromegaly, no chronic conditions, and no regular medication use. Patients without accessible hospital records were excluded. All participants provided written informed consent after receiving comprehensive information about the study. The study adhered to the principles of the Declaration of Helsinki.

Sample size calculation was performed using G*Power 3.1 software with an alpha level of 0.05 and statistical power of 80% [23]. For a large effect size, approximately 26 participants per group were required. The study included 36 patients in the acromegaly group, which meets the sample size requirement under this assumption.

### Laboratory measurements

After an overnight fasting period of no less than 8 h, venous blood samples for laboratory testing were collected between 8:00 and 10:00 AM. The parameters measured included levels of growth hormone (GH), insulin-like growth factor-1 (IGF-1), adrenocorticotropic hormone (ACTH), cortisol, thyroid-stimulating hormone (TSH), thyroxine (T4), fasting blood glucose concentration, HbA1c levels, and bone mineral density (BMD). Outcomes were meticulously documented. BMD measurements were conducted utilising dual-energy X-ray absorptiometry (DEXA) to evaluate the density of vertebral and femoral bones. GH, IGF-1, ACTH, cortisol, TSH, and T4 levels were quantified utilising Radioimmunoassay (RIA) methodology, while HbA1c levels were assessed through electrophoresis techniques. In patients not undergoing treatment with somatostatin receptor ligands, remission was defined as GH levels staying within the normal range., and the lowest GH level observed during a 75-gram oral glucose tolerance test (OGTT) without medication fell below 1 µg/L. In patients receiving SSRL, remission was defined as a normal IGF1 level. This subgroup of patients was classified as being in remission, while those not meeting these criteria were categorised as having persistent or recurrent disease.

### Data collection

Demographic information, comprising age, gender, and educational level, were systematically recorded. Clinical data such as disease status (remission, persistent, or recurrent), treatment modalities, history of surgery or radiotherapy, presence of panhypopituitarism, medications, and comorbidities were also collected.

Participants were provided with a 6-item questionnaire to complete. The initial section of the questionnaire gathered sociodemographic information, comprising age, gender, and level of education. The subsequent section evaluated participants’ oral hygiene practices, encompassing parameters such as the frequency of tooth brushing, flossing, tongue cleaning, mouthwash utilisation, frequency of dental check-ups, and consumption of sugary foods (refer to Appendix 1 for details). Each response in the questionnaire was assigned numerical values based on a devised scoring system, as outlined in Appendix 1. The total score ranged from 0 to 18, with higher scores indicative of better oral hygiene practices and lower scores suggesting poorer oral hygiene.

The oral hygiene questionnaire was newly developed for the purpose of this study. To ensure content validity, it was reviewed by two experts in oral and maxillofacial radiology and one expert in periodontology. A pilot test was conducted with 10 individuals from a population similar to the study sample to assess the clarity and internal consistency of the items. Based on the feedback, minor modifications were made to improve comprehension and item relevance. The finalised questionnaire consisted of six items, with total scores ranging from 0 to 18.

### Oral examination

All oral examinations were conducted by a single examiner, a Professor in the Department of Oral Diagnosis and Maxillofacial Radiology with clinical experience since 2002. Examinations took place in the dental clinic unit of Ankara City Hospital under adequate illumination, utilising a dental mirror, dental probe, and a North Carolina 15 periodontal probe. Oral health parameters were systematically recorded, including dental caries, periodontal health, and presence of removable and fixed prostheses. Additionally, participants were asked about smoking, alcohol consumption, and xerostomia (dry mouth).

The DMFT (decayed, missing, filled teeth) index was used to assess dental caries [10–24]. The DMFT index is calculated using the formula: DMFT = (D + M + F)/T, where D denotes decayed teeth, M denotes missing teeth, F denotes filled teeth, and T denotes the total number of individuals examined.

Clinical periodontal health was assessed using the periodontal screening index (PSI) [11–25] and plaque index (PI) [12–26] scores. The PSI scoring system stages periodontal tissues from 0 to 4, with stage 0 representing periodontally healthy tissue, stages 1 and 2 indicating gingivitis, and stages 3 and 4 indicating periodontitis (inflammation of the gum and supporting alveolar bone). The PI serves as an objective clinical assessment tool for oral hygiene, scoring plaque accumulation caused by food debris on tooth surfaces from 0 to 3, with lower values indicating clean teeth and higher values indicating uncleaned teeth. All measurements were conducted using a periodontal probe on one premolar and one molar tooth in each quadrant of the upper and lower jaws.

Although oral indices such as DMFT, PSI, and PI were the primary focus relevant to the study aims, other oral findings, including removable and fixed prostheses, and pericoronitis, were also documented to provide a comprehensive overview of oral health status. These findings were reported descriptively and were not included in the main statistical analyses as they lie outside the primary objectives.

Intra-rater reliability was evaluated by re-examining 20% of participants after a two-week interval. The Intraclass Correlation Coefficient (ICC) was calculated for continuous variables (DMFT, PSI, and PI scores), showing excellent reliability with ICC values above 0.85. Cohen’s Kappa was used for categorical variables, indicating substantial agreement (kappa > 0.75). Due to the single-examiner design, inter-rater reliability was not applicable.

### Statistical analysis

All statistical analyses were carried out using IBM SPSS Statistics version 20 (Chicago, IL, USA). Continuous variables are summarized as mean ± standard deviation, as well as median, minimum, and maximum values, while categorical variables are expressed as frequencies and percentages. The Shapiro–Wilk test was applied to determine the normality of continuous data distributions. Comparisons between patients and controls were performed with an independent samples t-test for normally distributed variables and with a Mann–Whitney U test for skewed distributions. When comparisons involved more than two groups, the Kruskal–Wallis test was used. Associations between categorical variables were examined with Chi-square or Fisher’s Exact tests, as appropriate. Relationships between continuous variables were assessed using Spearman’s rank correlation. In addition, univariate logistic regression analyses were conducted to explore potential risk factors related to macroglossia and xerostomia. Although the control group size was smaller than that of the acromegaly group, appropriate statistical methods were used to address this imbalance. Specifically, non-parametric tests such as the Mann-Whitney U test and Kruskal-Wallis analysis were employed, as they are less sensitive to unequal sample sizes and non-normal distributions. This approach ensured reliable comparisons despite the group size discrepancy. Statistical significance was set at *p* < 0.05.

## Results

The study included 35 patients with acromegaly and 20 healthy controls. The mean age of patients was 51.37 ± 9.59 years, with no significant difference compared to controls (*p* > 0.05). The average disease duration was 11.80 ± 7.07 years. Among patients, 45.7% were female, 54.3% male, and 88.6% were married. Over half of the patients (54.3%) were university graduates. Clinical characteristics are summarised in Table 1.

**Table 1:** Clinical characteristics of patients

*n* = 35	Mean ± SD	
IGF-1	219.71 ± 108.50	
GH (*n* = 34)	1.91 ± 2.67	
	**n**	**%**
Remission		
In remission	29	82.9
Persistent/Recurrent	6	17.1
Surgery		
Absent	2	5.7
Transsphenoidal (once)	26	74.3
Transsphenoidal (twice)	7	20.0
Use of somatostatin		
Absent	17	48.6
Present	18	51.4
Types of SRL		
Absent	17	48.6
Octreotide	10	28.6
Lanreotide	5	14.3
Octreotide + Cabergoline	3	8.6
Radiotherapy		
Absent	32	91.4
Present	3	8.6

*n* number of subjects, *SD* Standard deviation, *IGF*-*1* Insulin-like growth factor-1, *GH* Growth hormone

Most patients (82.9%) achieved remission. Macroadenomas were present in 88.6%, and 51.4% used somatostatin analogues. Comorbidities included Diabetes Mellitus (37.1%), hypothyroidism (37.1%), coronary artery disease (22.9%), hypertension (20%), adrenal insufficiency (14.3%), gonadal insufficiency (5.5%), and panhypopituitarism (5.7%).

Laboratory findings revealed significantly lower TSH levels in patients compared to controls (*p* < 0.05), while T4 levels showed no significant difference (*p* > 0.05) (Table 2). HbA1c and fasting glucose levels were significantly higher in patients than controls (*p* < 0.01 and *p* < 0.05, respectively), with no differences in creatinine levels (*p* > 0.05).

**Table 2:** Comparisons of age, gender, TSH, T4, HbA1c, Fasting-Glucose, creatinine values in patient and control groups

	Patient		Control		*p*
	Mean ± SD		Mean ± SD		
Age	51.37 ± 9.59		47.55 ± 5.08		0.059
TSH	1.54 ± 1.03		2.11 ± 0.96		**0.034**
T4	1.17 ± 0.24		1.17 ± 0.14		0.624
HbA1c	6.09 ± 0.84		5.52 ± 0.34		**0.002**
Fasting Blood Glucose	103.54 ± 27.55		88.15 ± 11.01		**0.020**
Creatinine	0.79 ± 0.18		0.80 ± 0.12		0.753
	**n**	**%**	**n**	**%**	
Gender					
Female	16	45.7	10	50	0.759
Male	19	54.3	10	50	

*SD* Standard deviation, *TSH* Thyroid-stimulating hormone, *T4* Thyroxine, *HbA1c* Glycated hemoglobin, *n* Noun

Oral hygiene scores and DMFT values showed no significant differences between groups (*p* > 0.05) (Table 3). However, tongue lesions, particularly macroglossia (82.9%) and fissured tongue (51.4%), were significantly more prevalent in patients compared to controls (*p* < 0.001) (Table 4). No tongue lesions were detected in controls.

**Table 3:** Comparison of Oral-Hygiene scores and DMFT scores in patient and control groups

	Patient	Control	*p*
	Mean ± SD	Mean ± SD	
Oral Hygiene Score	6.60 ± 3.29	7.30 ± 2.90	0.443
DMFT	14.43 ± 10.18	12.60 ± 8.69	0.611

Oral Hygiene Score: Each response was scored according to a defined system (see Appendix 1). Total scores ranged from 0 to 18, with higher scores indicating better oral hygiene practices and lower scores reflecting poorer oral hygiene

*SD* Standard deviation, *DMFT* Decayed, missing, and filled teeth index

**Table 4:** Comparison of oral lesion findings in patient and control groups

	Patient		Control		*p*
	*n*	%	*n*	%	
Macroglossia					
Absent	6	17.1	20	100	**< 0.001**
Present	29	82.9	0	0	
Fissured Tongue					
Absent	17	48.6	20	100	**< 0.001**
Present	18	51.4	0	0	
Black Hairy Tongue					
Absent	30	85.7	20	100	0.147
Present	5	14.3	0	0	
Crenated Tongue					
Absent	33	94.3	20	100	0.529
Present	2	5.7	0	0	
Varicose					
Absent	34	97.1	20	100	1.000
Present	1	2.9	0	0	
Tongue lesion					
Absent	6	17.1	20	100	**< 0.001**
Present	29	82.9	0	0	
Angular Cheilitis					
Absent	32	91.4	20	100	0.293
Present	3	8.6	0	0	
Actinic Cheilitis					
Absent	32	9.4	20	100	0.293
Present	3	8.6	0	0	
Macrochelia					
Absent	34	97.1	20	100	1.000
Present	1	2.9	0	0	
Lip Lesion					
Absent	31	88.6	20	100	0.285
Present	4	11.4	0	0	
Buccal Lesion					
Absent	33	94.3	18	90.0	0.616
Present	2	5.7	2	10.0	
Other Lesion					
Absent	31	88.6	20	100	1.000
Present	4	11.4	0	0	

*n* Noun

The Periodontal Screening Index (PSI) and plaque index scores did not differ significantly between groups (*p* > 0.05) (Table 5). Similarly, the prevalence of dry mouth, smoking, and alcohol use showed no significant differences (*p* > 0.05).

**Table 5:** Comparison of periodontal screening index, plaque index, dry mouth, smoking and alcohol findings in patient and control groups

	Patient		Control		*p*
	*n*	%	*n*	%	
Periodontal Screening Index					
0	7	20.0	0	0	0.061
1	8	22.9	3	15.0	
2	8	22.9	5	25.0	
3	8	22.9	11	55.0	
4	4	11.3	1	5.0	
Plaque Index					
0	3	8.6	0	0	0.200
1	10	28.6	2	10.0	
2	11	31.4	9	45.0	
3	11	31.4	9	45.0	
Xerostomia					
Absent	17	48.6	12	60.0	0.414
Present	18	51.4	8	40.0	
Smoking					
Absent	24	68.6	9	45.0	0.086
Present	11	31.4	11	55.0	
Alcohol					
Absent	34	97.1	16	80.0	0.053
Present	1	2.9	4	20.0	

*n* Noun

In patients, DMFT scores did not differ significantly based on remission status, dry mouth, surgical history, somatostatin use, radiotherapy, comorbidities, or smoking status (*p* > 0.05 for all comparisons) (Table 6).

**Table 6:** Comparison of DMFT scores in patients according to independent variables

		DMFT	*p*
	*n*	Mean ± SD	
Xerostomia			
Absent	17	14.59 ± 11.44	0.909
Present	18	14.28 ± 9.17	
Remission			
In Remission	29	13.66 ± 10.01	0.334
Persistent/Recurrent	6	18.17 ± 11.10	
Surgical			
Once Transsphenoidal	26	15.08 ± 11.08	0.560
Twice Transsphenoidal	7	10.86 ± 5.92	
Somatostatin Use			
Absent	17	11.71 ± 8.83	0.134
Present	18	17.00 ± 10,93	
Radiotherapy			
Absent	32	14.47 ± 10.51	0.890
Present	3	14.00 ± 7.00	
DM			
Absent	22	12.68 ± 8.82	0.287
Present	13	17.38 ± 11.94	
Hypothyroidism			
Absent	22	13.32 ± 10.59	0.287
Present	13	16.31 ± 9.56	
Coronary Artery Disease			
Absent	27	13.67 ± 9.63	0.550
Present	8	17.00 ± 12.22	
Hypertension			
Absent	28	13.54 ± 9.80	0.320
Present	7	18.00 ± 11.67	
Adrenal Insufficiency			
Absent	30	14.97 ± 10.59	0.536
Present	5	11.20 ± 7.29	
Gonadal Insufficiency			
Absent	33	14.97 ± 10.24	0.188
Present	2	5.50 ± 0.70	
Panhypopituitarism			
Absent	33	14.97 ± 10.24	0.188
Present	2	5.50 ± 0.70	
Smoking			
Absent	24	12.58 ± 9.73	0.099
Present	11	18.45 ± 10.42	

*DMFT* Decayed, missing, and filled teeth index, *n* Noun, *SD* Standard deviation

Correlation analysis revealed no significant associations between DMFT scores and disease duration, IGF-1, GH, cortisol, ACTH levels, oral hygiene, plaque index, or PSI scores (*p* > 0.05). A negative correlation was observed between oral hygiene scores and PSI scores (*p* < 0.05), and a positive correlation between plaque index and PSI scores (*p* < 0.001) in patients (Table 7). No significant correlations were found among oral hygiene, plaque index, PSI, or DMFT scores in controls (*p* > 0.05) (Table 8). DMFT scores did not differ significantly in controls with or without dry mouth (*p* > 0.05) (Table 9).

**Table 7:** Correlations between disease duration, IGF-1, GH, cortisol, ACTH values, oral hygiene, plaque index PSI scores and DMFT in patients

	DMFT		PSI	
	*r*	*p*	*r*	*p*
Disease Duration	−0.039	0.824	0.039	0.824
IGF-1	−0.018	0.918	0.158	0.366
GH	−0.250	0.154	0.021	0.904
ACTH	0.282	0.154	0.005	0.979
Cortisol	0.094	0.604	−0.058	0.749
Oral Hygiene Score	−0.195	0.261	**−0.414**	**0.013**
Plaque Index	−0.108	0.538	**0.807**	**< 0.001**
PSI	0.027	0.879	-	-

*DMFT* Decayed, missing, and filled teeth index, *PSI* Periodontal Screening Index, *IGF*-*1* Insulin-like growth factor-1, *GH* Growth hormone, *ACTH* Adrenocorticotropic hormone, Oral Hygiene Score Each response was scored according to a defined system (see Appendix 1)

Total scores ranged from 0 to 18, with higher scores indicating better oral hygiene practices and lower scores reflecting poorer oral hygiene

**Table 8:** Correlations between oral hygiene score, plaque index, PSI scores and DMFT in individuals in the control group

	DMFT		PSI	
	*r*	*p*	*r*	*p*
Oral Hygiene Score	−0.269	0.252	−0.313	0.179
Plaque Index	−0.291	0.214	0.314	0.177
PSI	−0.028	0.906	-	-

*DMFT* Decayed, missing, and filled teeth index, *PSI* Periodontal Screening Index, Oral Hygiene Score: Each response was scored according to a defined system (see Appendix 1)

Total scores ranged from 0 to 18, with higher scores indicating better oral hygiene practices and lower scores reflecting poorer oral hygiene

**Table 9:** Comparison of DMFT scores of individuals in the control group with those without dry mouth

		DMFT	
	*n*	Mean ± SD	*p*
Xerostomia			
Absent	12	10.42 ± 8.40	0.135
Present	8	15.88 ± 8.59	

## Discussion

This study aims to investigate the relationship between oral mucosal lesions, disease activity, and laboratory parameters in acromegaly patients. It is the first study in the literature comparing oral hygiene, dental scores, and lesions in acromegaly patients with a control group and exploring their relationship with disease parameters. By examining the frequency and variety of oral mucosal lesions in acromegaly patients, this study seeks to evaluate the potential impact of these lesions on the disease prognosis.

In our study, the mean age of the patients was 51.37 ± 9.59 years, with a minimum age of 31 and a maximum age of 65. The duration of the disease averaged 11.80 years with a standard deviation of 7.07, with a minimum duration of 0.3 years and a maximum of 25 years. In a study conducted in Turkey, a total of 547 acromegaly patients from 10 different centres were included (Gender, n (%): female, 300 (55%); male, 247 (45%)). The median follow-up duration was 8 years (range 0–32), the median age at diagnosis was 41 years (range 16–76), and the median duration of diagnosis was 48 months (range 1–300) [13–27]. When compared to previous literature, our study suggests that the age at diagnosis is younger. This difference is thought to be attributed to easier access to tertiary healthcare and endocrinology specialists in our country compared to other countries. Furthermore, 54.3% of the patient group in our study were university graduates. This situation was also considered a factor contributing to the early age at diagnosis. In our study, 45.7% of the patients were female, and 54.3% were male, which is consistent with the literature [14–28].

Among the 35 patients who participated in our study, 26 underwent transsphenoidal surgery once, and 7 were treated with recurrent transsphenoidal surgery. Two patients did not undergo surgery due to high anaesthesia risk attributed to comorbid conditions. All patients who underwent surgery were operated on using the transsphenoidal method. In our study, 82.9% of the patients were observed to be in remission, 31 patients (88.6%) were diagnosed with macroadenoma, and 4 patients (11.4%) with microadenoma. It was observed that the remission rate at our centre was higher compared to previous reports ([15]– [16] [29]– [30]). In our study, 18 patients (51.4%) were using somatostatin receptor ligands. Out of these, 10 patients were using octreotide LAR (28.6%), 5 patients were using lanreotide LAR (14.3%), and 3 patients were using a combination of octreotide and cabergoline (8.6%). Similar side effects have also been mentioned in the literature [17–31]. In our study, only 3 patients (8.6%) received radiotherapy. Among these patients, one (2.8%) underwent conventional type radiotherapy, while the other two (5.7%) received stereotactic radiotherapy. For individuals with acromegaly unresponsive to surgical or medical therapy, radiotherapy continues to serve as an effective modality in cases of residual tumor and failure to achieve biochemical remission. However, concerns still exist regarding the delayed biochemical effect and potential toxicity of radiation therapy, particularly given the high rates of hypopituitarism. Stereotactic radiotherapy is currently more preferred, yielding more favourable results in terms of side effects and treatment success compared to conventional radiotherapy. Despite its effectiveness, there are ongoing debates and concerns about the necessity and potential toxicity of radiotherapy, making its use a subject of discussion [18–32].

In our study, 13 patients (37.1%) had diabetes mellitus (DM). Diabetes mellitus is one of the common complications of acromegaly. In a study, the prevalence of diabetes mellitus in acromegaly patients was reported to be 12–37% [19–33]. Compared to the literature ([19, 20] [33]– [34]), our study identified a higher prevalence of diabetes mellitus in the patient group. The HbA1c values of the patients were found to be significantly higher than those of the individuals in the control group. The mean HbA1c value for the patient group was 6.09 ± 0.84, falling within normal ranges. However, this statistically significant result was not clinically significant.

In acromegaly, hypertension represents a major comorbidity that contributes to higher morbidity and mortality. The frequency of hypertension is estimated at about 35%, though values between 18% and 60% have been described in various clinical series [21–34]. The blood pressure of these patients is regularly monitored under various antihypertensive treatments. Xerostomia has been specifically reported in relation to the use of ACE inhibitors and diuretics within antihypertensive therapy. Etiologically, drugs have been identified as the most common cause of secondary xerostomia [22–36]. Among the 7 hypertensive patients in our study, 3 had xerostomia, and they were using ACE inhibitors. It is speculated that the use of antihypertensive medications causing xerostomia may have negatively affected the dental health of these patients. Moreover, the evaluation of xerostomia was based on self-reported symptoms without measurement of unstimulated or stimulated salivary flow rate, which may have influenced the accuracy of the findings. Future studies should incorporate objective measurements of salivary flow rate to validate self-reported xerostomia and minimise information bias.

In our study, 5 patients (14.3%) had secondary adrenal insufficiency, and these patients were receiving prednisolone treatment. In a study [24–37] comparing the oral health of 100 patients receiving long-term corticosteroid therapy for at least 3 months with age and gender-matched 100 healthy controls, the clinical and radiological effects of corticosteroid therapy on oral health were evaluated. A significantly higher level of Candida was found in steroid-using patients. Additionally, bone density in the study group was significantly lower compared to the control group [24–37]. The TSH values of the patients were significantly lower than those of the individuals in the control group. The mean TSH value in the patient group was 1.54 ± 1.03, within normal limits. Although statistically significant, this finding was not clinically significant.

In our study, macroglossia was present in 29 patients (82.9%). A significant difference was found in the rates of macroglossia between the patients and individuals in the control group (*p* < 0.001). Macroglossia is an expected clinical condition in acromegaly patients. In acromegaly, the increased occurrence of macroglossia may be explained by sustained elevations in GH and IGF-1. These hormones stimulate soft tissue proliferation, collagen synthesis, and extracellular matrix accumulation, leading to generalised tissue hypertrophy. In the oral cavity, this manifests as enlargement of the tongue [25–38]. A review study revealed that macroglossia was observed in 54% to 58% of acromegaly patients [26–39]. Macroglossia increases the risk of sleep apnea in affected individuals [27–40]. Patients with severe obstructive sleep apnea show greater vulnerability to overall and cardiovascular-related mortality [28–41]. In this present study, the statistical analysis results indicated that the sole determinant of the presence of macroglossia was the presence of acromegaly. Univariate Logistic Regression analysis revealed that factors such as disease duration, IGF-1, GH, ACTH, cortisol, remission status, surgery, somatostatin use, diabetes mellitus (DM), hypothyroidism, and adrenal insufficiency were not identified as effective risk factors for macroglossia. It was observed that macroglossia persisted even in patients in remission.

The observed increase in macroglossia and fissured tongue among patients with acromegaly can be attributed to the chronic exposure to elevated levels of GH and IGF-1. These hormones stimulate soft tissue proliferation, collagen synthesis, and extracellular matrix accumulation, leading to generalised tissue hypertrophy. In the oral cavity, this manifests as enlargement of the tongue (macroglossia), which may subsequently develop fissures due to mechanical irritation and surface dehydration. These findings are consistent with the known systemic effects of GH/IGF-1 excess and reflect the disease’s influence on both soft and hard tissue structures in the craniofacial region.

Lingua plicata, or fissured tongue, generally presents without symptoms and manifests as grooves and furrows of varying depth on the dorsal tongue. Published data indicate a prevalence in the range of 10–20% [29–42]. Fissured tongue has been associated with several factors, including hereditary traits, oral candida, and the use of smokeless tobacco [43–46]. It is also observed in systemic conditions such as hypertension, psoriasis, orofacial granulomatosis, and diabetes mellitus. In diabetic individuals, poor glycemic control, impaired immune responses, decreased blood circulation, dry mouth, and alterations in both the quantity and quality of saliva may contribute to the development of fissured tongue [47–49]. In our study, fissured tongue was observed in 18 patients (51.4%). There was a significant difference in the prevalence of fissured tongue between the patients and the control group (*p* < 0.001). The literature does not commonly report the prevalence of fissured tongue in acromegaly patients. Our study systematically evaluated other tongue lesions identified during examinations. When all tongue lesions were considered, lesions were observed in 29 patients (82.9%). No tongue lesions were found in the control group participants. Studies in the literature ([30]– [31] [50]– [51]) examining oral mucosal changes in acromegaly patients have typically focused on macroglossia, neglecting soft tissue changes such as fissured tongue, varices, angular cheilitis, and actinic cheilitis. The results of this study provide valuable information about soft tissue changes affecting the jaw and facial regions in acromegaly patients. This study is the first in the literature to demonstrate detailed oral mucosal changes in acromegaly patients. In our study, varices were found in one patient, angular cheilitis (cracks at the corners of the mouth due to infection) in three patients, and actinic cheilitis, a premalignant lesion, in three patients. In light of this study, it is evident that the impact on the tongue is not limited to macroglossia, emphasising the importance of a thorough examination of oral lesions in acromegaly patients by both endocrinologists and, if necessary, dentists.

Human teeth constitute a highly valuable organ system that plays a crucial role in grasping and processing food. Defined as a localized lesion of the hard tissues of the teeth, dental caries develops from acid production during microbial fermentation of ingested carbohydrates [32–52]. Dental caries is recognised by the World Health Organisation as the most prevalent health issue globally [33–53]. The risk factors for dental caries include a high-carbohydrate diet, especially sucrose, and/or low saliva secretion. The oral hygiene questionnaire used in our study not only queried patients about their oral hygiene habits but also included questions about the frequency of sugar consumption. When carbohydrates are fermented by oral microorganisms (Streptococcus mutans), the pH of saliva drops, and the acidic substrates produced as a result of fermentation lead to demineralisation of dental hard tissues and, consequently, dental caries [34–54]. Adequate oral hygiene ensures the removal of food residues from dental surfaces, eliminating the primary risk for caries, which is carbohydrate fermentation. Tooth indexes, individuals or communities are defined as tools used for the identification and recording of dental caries [35–55]. An ideal index is expected to be simple, objective, valid, reliable, measurable, repeatable, sensitive, and acceptable [36–56]. Since its introduction in 1938 and subsequent recommendation by the World Health Organization (WHO), the DMFT (decayed, missing, filled teeth) index has been the most frequently employed index in epidemiological investigations. DMFT index is a measure representing the total number of an individual’s decayed, missing, and filled teeth, first defined by Klein and Palmer in 1938 [10–24]. In 1939, Bödecker proposed the DMFS (decayed, missing, filled surface) index by suggesting the calculation of the surface count instead of the tooth count. This index represents the total number of an individual’s decayed, missing, and filled tooth surfaces [37–57]. The primary goal of this index system is to provide a meaningful comparison of the decayed status across various populations [38–58]. Immune system disorders, dysfunction of salivary glands, diabetes mellitus, and the use of various medications causing dry mouth increase susceptibility to dental caries [39–59]. In our study, a comparison of DMFT scores was conducted based on independent variables that could contribute to caries susceptibility, such as remission status, xerostomia, somatostatin use, the number of surgeries, radiotherapy treatment, diabetes mellitus, hypothyroidism, coronary artery disease, hypertension, panhypopituitarism, adrenal insufficiency, and gonadal insufficiency. No statistically significant results were found. In a meta-analysis study, diabetes mellitus (DM) and patients with poor glycemic control were evaluated using the DMFT scoring system. The findings showed a higher average DMFT score in individuals with diabetes compared to healthy controls. In individuals with Type 2 DM, three times more root caries were observed compared to non-diabetic individuals. Among the diabetic population, individuals with uncontrolled glycemic levels exhibited a higher caries prevalence compared to controlled diabetic individuals [40–60].

In a study, the average prevalence of radiation caries was reported as 28.1%. In this context, the mean DMFT of irradiated patients was found to be 9.19 [41–61]. In a study conducted by Konjhodzic-Prcic and colleagues in 2010, the DMFT index was used, and they found that the incidence of radiation caries increased from 19.4 to 23.9 after 6 months of radiation therapy [42–62]. In our study, three patients received radiation therapy, and their DMFT scores were 7, 14, and 21, respectively. In a study conducted in 2016, the relationship between thyroid dysfunction and DMFT in children was examined. The study compared 100 healthy individuals with 100 individuals with thyroid dysfunction. Although the DMFT and scores were found to be higher in the group with thyroid dysfunction compared to the control group, the score difference was not statistically significant [43–63].

According to the results of our study, there was a statistically significant relationship between the DMFT index and oral hygiene scores, as well as the plaque index scores, which quantitatively evaluate oral hygiene. The findings of our study suggest that dental caries in acromegaly patients is not attributed to acromegaly and comorbidities but is rather associated with poor oral hygiene. This aligns with findings from Roumeau et al. (44 − 11), who reported comparable DMFT values in acromegalic patients and the general French population, with the noteworthy distinction that acromegaly patients had more filled and fewer missing teeth. These observations were interpreted as indicators of better dental care utilisation among acromegaly patients, possibly related to socioeconomic factors. In our cohort, the presence of dental caries was significantly associated with poor oral hygiene, as evidenced by the correlation between DMFT scores and both plaque index and oral hygiene questionnaire results. However, this correlation was absent in the control group, suggesting that patients with acromegaly may be more prone to caries due to challenges in maintaining oral hygiene—potentially exacerbated by anatomical changes like macroglossia and joint stiffness.

Periodontitis is a chronic inflammatory disease caused by gram-negative anaerobic bacteria, leading to gingival inflammation and destruction of both the supporting soft and hard tissues (periodontal tissues) surrounding the teeth. Bacterial plaque is the primary etiological factor associated with periodontitis, but there are several other variables that may increase the risk of developing the disease. Two of these variables are definitively identified as risk factors: tobacco use and diabetes [45–64]. The Periodontal Screening Index (PSI) provides information about the periodontal condition and offers insights into the necessary treatment [46–65]. In a study [47]. Examining the relationship between periodontitis and diabetes mellitus, 111 non-diabetic (ND), 101 type 1 diabetic (T1D), and 236 type 2 diabetic (T2D) patients were included. The study compared DMFT and Periodontal Screening Index (PSI) among the study groups. According to the results of the previous study, PSI scores were significantly higher in T2D and T1D patients compared to non-diabetic patients, and periodontitis was detected in approximately 90% of T2D patients [47–66]. We found a significant association between PSI scores and both plaque index and oral hygiene, but no correlation with hormonal parameters or disease duration. These findings suggest that, similar to the general population, bacterial plaque remains the central etiological factor for periodontal disease in acromegaly [48–67]. Roumeau et al. (44 − 11) also observed a relatively low prevalence of periodontitis in their acromegaly cohort and proposed a protective role of GH and IGF-1 on soft tissues, potentially linked to the presence of a thick gingival biotype. In their study, 9 of 29 patients exhibited thick gingival tissues, which are associated with reduced periodontal vulnerability. While we did not assess gingival biotype in our study, the absence of severe periodontitis supports the hypothesis that hormonal influences may confer some level of periodontal resilience in acromegaly patients [49–68].

The effects of acromegaly on gingival and periodontal tissues are still not fully understood to this day. Our study represents the first examination of the relationship between acromegaly and periodontitis in the literature. Acromegaly itself does not cause immune dysfunction. However, concomitant comorbid conditions such as adrenal insufficiency, diabetes mellitus, and hypothyroidism increase susceptibility to infection. Nevertheless, according to our study results, there was no significant relationship between the Periodontal Screening Index (PSI) and disease duration, IGF-1, GH, cortisol, and ACTH. Our findings indicate a significant association between PSI and plaque index as well as oral hygiene scores in acromegaly patients. As mentioned above, bacterial plaque serves as the fundamental etiological factor for periodontitis. In acromegalic patients, the typical association between poor oral hygiene and periodontal disease may be modulated by disease-specific factors such as altered bone metabolism, thickened gingival biotype, and soft tissue overgrowth. GH and IGF-1 excess contribute to increased collagen synthesis and fibroblast proliferation, potentially enhancing gingival volume and resilience. These changes may either predispose to or protect against periodontal damage, depending on the balance between tissue remodelling and microbial insult. Future studies should include clinical evaluation of the gingival biotype to better understand its potential protective role against periodontal disease in patients with acromegaly. Furthermore, macroglossia in acromegalic patients may limit effective access to posterior oral regions during toothbrushing, thereby impairing mechanical plaque removal and contributing to poor oral hygiene despite adequate awareness.

This study has several limitations. The relatively small sample size and cross-sectional design may limit the generalizability of the findings. The subjective nature of xerostomia evaluation and the use of self-reported oral hygiene questionnaires may introduce information bias. Additionally, single-point assessments preclude longitudinal analysis of oral health changes. Despite these limitations, the study is strengthened by its comprehensive evaluation of oral and systemic parameters and by the inclusion of a control group for comparative analysis. Future research should include longitudinal studies to track the progression of oral health in patients with acromegaly over time and to investigate the potential impact of treatment duration and disease activity on oral findings. Additionally, incorporating objective salivary flow measurements, assessing the effect of macroglossia on oral hygiene ability, and utilising salivary or imaging biomarkers could provide deeper insights into the mechanisms underlying oral health deterioration in this population.

## Conclusions

Our findings showed that macroglossia and fissured tongue were significantly more prevalent in the patient group than in the control group, and oral mucosal lesions do not directly affected by the course or prognosis of acromegaly. Results of this study revealed that acromegaly itself may pose an independent risk for periodontal disease. Therefore, patients with acromegaly must prioritise not only their systemic health but also their oral health. Improved oral hygiene practices and a collaborative approach between endocrinologists and dental professionals are essential for preventing both systemic and oral complications in this complex disorder.

## Data Availability

The datasets used and/or analysed during the current study are available from the corresponding author, **Elif Yildizer** (email: dryildizerelif@gmail.com), upon reasonable request.

## Appendix

### Questionnaire Form

Age:

Gender:

Occupation - Education Status:

Patient’s Oral Hygiene Habits.

1. How many times a year do you visit the dentist?

**Table:**

None (0p)	1 (1p)	2 (2p)	3 (3p)	> 3 (4p)

2. How many times a day do you brush your teeth?

**Table:**

None (0p)	1 (1p)	2 (2p)	3 (3p)	> 3 (4p)

3. Which products do you use to maintain your oral hygiene?

**Table:**

None (0p)	Toothbrush (1p)	Toothpaste (1p)	Mouthwash (1p)

4. Do you perform interdental cleaning? Which products do you use?

**Table:**

No (0p)	Yes		
	Interdental brush(1p)	Dental floss(1p)	Toothpick (1p)

5. Do you have a tongue cleaning habit?

**Table:**

No (0p)	Yes (1p)

6. How often do you eat sugary foods?

**Table:**

Rarely (3p)	Two to three times a week (2p)	Once a day (1p)	Every day without fail (0p)

## Abbreviations

ACE: Angiotensin-converting enzyme
ACTH: Adrenocorticotropic hormone
BMD: Bone mineral density
CAD: Coronary artery disease
DEXA: Dual-energy X-ray absorptiometry
DM: Diabetes mellitus
DMFS: Decayed, missing, filled surfaces index
DMFT: Decayed, missing, filled teeth index
GH: Growth hormone
HbA1c: Glycated hemoglobin
ICC: Intraclass correlation coefficient
IGF-1: Insulin-like growth factor-1
OGTT: Oral glucose tolerance test
PI: Plaque Index
PSI: Periodontal Screening Index
RIA: Radioimmunoassay
SD: Standard deviation
SPSS: Statistical Package for the Social Sciences
SRL: Somatostatin receptor ligand
T1D: Type 1 diabetes
T2D: Type 2 diabetes
T4: Thyroxine
TSH: Thyroid-stimulating hormone
WHO: World Health Organization
